# Assessment of Depression, Anxiety and Apathy in Prodromal and Early Huntington Disease

**DOI:** 10.1371/currents.RRN1242

**Published:** 2011-06-17

**Authors:** Anthony L Vaccarino, Terrence Sills, Karen E. Anderson, Anne-Catherine Bachoud-Lévi, Beth Borowsky, David Craufurd, Kevin Duff, Joseph Giuliano, Mark Groves, Mark Guttman, Peter Kupchak, Aileen K Ho, Jane S. Paulsen, Kenn Freddy Pedersen, Erik van Duijn, Daniel P van Kammen, Ken Evans

**Affiliations:** ^*^Research Methods, Ontario Cancer Biomarker Network, Toronto, Ontario, Canada; ^‡^Department of Psychiatry and Department of Neurology, University of Maryland, School of Medicine, Baltimore, MD USA; ^§^AP-HP, centre de référence maladie de Huntington; INSERM U955 E01- Ecole Normale Supérieure-University Paris Est, Neuropsychologie interventionnelle laboratory,Créteil- Paris, France; ^¶^Translational Medicine, CHDI Foundation, Inc., Princeton NJ; ^#^University of Manchester, Manchester Academic Health Sciences Centre and Central Manchester University Hospitals NHS Foundation Trust, Manchester, UK; ^**^Neurology, University of Utah, Salt Lake City UT USA; ^††^CHDI Foundation, Inc.; ^‡‡^Departments of Neurology and Psychiatry, Beth Israel Medical Center, New York, NY; ^§§^Division of Neurology, Department of Medicine, University of Toronto, Toronto, Ontario Canada; ^##^School of Psychology and Clinical Language Sciences, University of Reading, U.K.; ^***^Department of Psychiatry, The University of Iowa Carver College of Medicine, Iowa City, IA, USA; ^†††^The Norwegian Centre for Movement Disorders, Stavanger University Hospital, Stavanger, Norway & Department of Neurology, Stavanger University Hospital, Stavanger, Norway; ^‡‡‡^Department of Psychiatry, Leiden University Medical Centre, Leiden, Netherlands and ^§§§^CNS Drug Development consultant

## Abstract

The Functional Rating Scale Taskforce for pre-Huntington Disease (FuRST-pHD) is a multinational, multidisciplinary initiative with the goal of developing a data-driven, comprehensive, psychometrically sound, rating scale for assessing symptoms and functional ability in prodromal and early Huntington disease (HD) gene expansion carriers. The process involves input from numerous sources to identify relevant symptom domains, including HD individuals, caregivers, and experts from a variety of fields, as well as knowledge gained from the analysis of data from ongoing large-scale studies in HD using existing clinical scales. This is an iterative process in which an ongoing series of field tests in prodromal (prHD) and early HD individuals provides the team with data on which to make decisions regarding which questions should undergo further development or testing and which should be excluded. We report here the development and assessment of the first iteration of interview questions aimed to assess Depression, Anxiety and Apathy in prHD and early HD individuals.

## Introduction

Earliest clinical manifestations of Huntington disease (HD) are poorly characterized, and there is a need for clinical scales specifically designed to measure early symptoms in HD gene expansion carriers. The Functional Rating Scale Taskforce for pre-Huntington Disease (FuRST-pHD) is a multinational, multidisciplinary collaboration to develop a valid functional rating scale to assess changes in symptom severity in HD gene expansion carriers who do not yet meet criteria for a formal clinical diagnosis (prodromal HD or prHD) or are early manifest.[1] Such a measurement tool is essential to better understand the earliest manifestations of HD, and to evaluate novel therapies early in the course of disease. 

FuRST-pHD has established an inclusive process using input from numerous sources, including prHD and early HD individuals, caregivers, and experts from a variety of fields, as well as from ongoing large-scale HD studies using existing clinical scales.[1] As part of the process, an inclusive series of “Working Groups” of individuals with clinical and/or scale development expertise have been established to review existing data and develop interview questions within the specific domain under study. Once these interview questions are developed, they are distributed to trained raters for beta testing in gene expansion carriers. This is an iterative process, in which changes or deletions (as appropriate) are made based on empirical evidence obtained during field testing; the modified questions are then tested during subsequent iterations so that the list can ultimately be winnowed to select optimal items for scale inclusion. 

Mood-related symptoms, including depression and apathy, are thought to be a prevalent component of HD that may be present prior to a formal diagnosis of the disease.[2] However, these symptoms are incompletely understood, and the presence of other manifestations (i.e., motor and cognitive) may confound assessment. Furthermore, tools used to assess depression in HD were developed for other populations (i.e., Hamilton Depression Rating Scale), and thus may be less appropriate for assessing symptoms in HD. We report here the development and assessment of the first iteration of interview questions aimed to determine which depression-related symptoms are relevant and necessary to measure in a prHD population.

## Methods 

Working group meetings of individuals with a broad range of relevant expertise were held to assess Depression and Anxiety (January 21–23, 2009, Toronto, Canada) and Apathy (January 14–15, 2010, New York City, USA). The working groups' charge were to review available evidence and provide input into development of interview questions to assess depression, anxiety and apathy in prHD. 

****

### Evidence Reviewed

#### Data Mining.

Although existing tools were not specifically designed to assess early manifestations in HD gene carriers, studies using such measures can nevertheless provide rich and useful information about the expression of symptoms in the target population, the differentiation of early changes from those expressed in advanced disease, or similar symptoms seen in other disorders. There are a number of ongoing studies investigating the symptomatology and progression of prHD and HD that are accessible to the FuRST pHD program, including PREDICT-HD and REGISTRY. Data assessing depression- and anxiety-related symptomtology in prHD were reviewed and considered by the working group in developing the interview questions:

- Beck Depression Inventory (PREDICT-HD and REGISTRY)
- Hamilton Depression Rating Scale (REGISTRY)
- Symptoms Checklist-90 (PREDICT-HD)
- Unified Huntington's Disease Rating Scale-Behavioral Subscale) (PREDICT-HD and REGISTRY)

In addition, items from a rating scale in development designed to assess depression and anxiety in Major Depressive Disorder [3] were field tested within the FuRST-pHD program, thus providing the working group additional data to consider in developing the interview questions. These included: 

**

*Interest in hobbies and pastimes, Interest in social activities with friends, Interest in social activities with family,* *Guilt, Self-esteem, Hopelessness,* *Depressed mood,* *Anxiety (N=259),* *Interest in accomplishments,* *General anhedonia,* *Hobbies and pastimes, Drive, Physical weakness, Emotional fatigue, Rumination (N=93).*

#### Patient and Companion Input.

The FDA views input from participants, caregivers and family members as an essential element in developing valid clinical assessment tools.[4] To ensure that the scale reflects concepts that are important from the participant's perspective, patient/companion focus groups were held to identify early changes experienced by HD gene expansion carriers. The focus groups were held in a number of countries using the local languages (France, Netherlands, United Kingdom, United States, Portugal, and Spain) with all participants (prHD, early HD, and companions) being asked a series of open-ended questions related to symptom occurrence in prHD. All focus group sites had IRB/EC approval and all participants provided informed consent. The following depression/anxiety-related symptoms/problems were reported by the focus groups and considered in developing the interview questions:

- Depression-related: *Changes in mood, More emotional, Control of emotions, Depression, Sadness, Crying*
- Guilt/Self-esteem/hopelessness: *Loss of confidence, Feeling of inferiority, Insecurity*
- Apathy and Anhedonia: *Burned out, Tiredness, Loss/lack of initiative, Problems getting started, Apathy, Loss of energy, Fatigability, Lack of motivation for usual activities, Reduced sexual activity, No emotion, Loss of interest in family, Postponing activities*
- Anxiety-related: *Anxiety, Anxiety about complaints, Anguish, Preoccupation with doing new things, Worrying, Worrying/anxiety about future, Nervous, Panic, Stress, Stress at work, Fear, Fear of phone, Sweating, Face tensed, Tense, Tremor inside body*

#### Expert Opinion and Experience of Participants.

In addition to reviewing existing data, working group participant experiences and opinion were also discussed.

### Development of Interview Questions 

Based on data mining, gene expansion carrier, caregiver, expert and literature input symptom domains and definitions are identified that are thought to be important to prHD. These diverse sources of information provided an excellent starting point for establishing which symptoms are important to participants. After review of existing data, relevant symptom domains were identified and interview questions were developed to assess specific symptoms within each cluster (and determine their severity) in prHD (Figure 1). 

**Figure:**
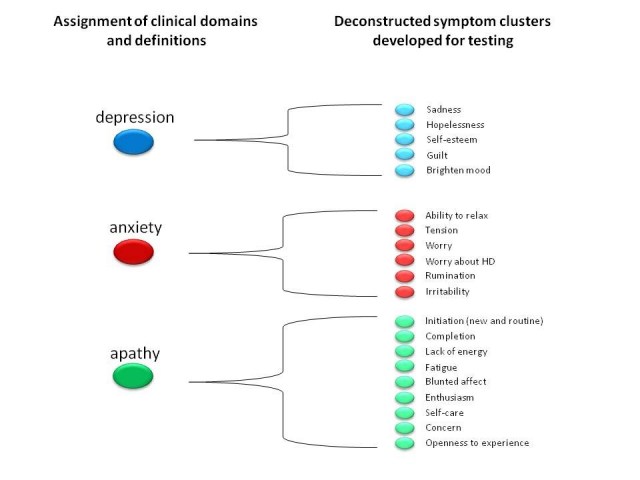
**Figure 1**. Based on patient, caregiver, expert, and literature input symptom domains are identified that are thought to be important to prHD. The Working Groups identify the constituent symptoms based on the words used by patients and caregivers, conventional definitions and their clinical and scientific opinion. Interview questions are then developed for each of the narrowly defined symptoms.

The FuRST-pHD has adopted a semi-structured clinician-administered interview similar to that used for the GRID-HAMD. The GRID format directs the rater to score symptom frequency and intensity separately, while giving them clear scoring anchors, a semi-structured interview guide, and overall definitions. This method has been employed successfully and is user-friendly, with acceptable agreement among independent raters.[5] The working group developed interview questions, including structured interview guides, scoring conventions, scoring anchors and symptom definitions. Following the meeting, draft interview questions were circulated for comment on a shared internet site (Sharepoint).

Based on a review of the evidence, 18 interview questions assessing depression, anxiety and apathy were developed for field testing (Table 1).

**Table 1.** Interview Questions

**Table:** 

**Interview Question**	**Description/definition**
**Sadness**	Assesses the degree to which an individual felt sad; should not be considered a global measure of depression.
**Brightening of mood**	Assesses the ease with which the individual can brighten their mood when sad or despondent.
**Drive-initiation of routine tasks**	Assesses the degree to which individuals need to push themselves to initiate routine tasks, irrespective of their level of physical energy, physical fatigue or anhedonia.
**Drive-initiation of enjoyable activities**	Assesses the degree to which individuals need to push themselves to initiate enjoyable activities, irrespective of their level of physical energy, physical fatigue or anhedonia.
**Drive-completion of tasks**	Assesses the degree to which individuals need to push themselves to complete tasks, regardless of physical energy level, physical fatigue or anhedonia, as well as how well you did the task.
**Lack of energy**	Assesses person’s experience of lack of energy/exhaustion (e.g., feeling drained, or exhausted, or empty, depleted).
**Tiredness/fatigue**	Assess effects of tiredness/fatigue in the performance of day to day routine activities.
**Blunting of affect**	Assesses the ability to react with adequate emotion to circumstances or people.
**Ability to relax**	Assesses ability to relax with normal stresses of life.
**Tension**	Assess inner tension that a person experiences.
**Excessive Worry**	Assesses worrying that is out of proportion in time spent or intensity of worry; should not include worrying associated with having HD.
**Worry about HD**	Assesses worrying associated with having HD.
**Confidence**	Assesses feeling of confidence in handing different situations or tasks.
**Rumination**	Assesses the occurrence of repetitive, intrusive, conscious, and aversive thoughts around some past event or decision that is regretted.
**Self-care**	Assesses the interest in maintaining personal care.
**Enthusiasm**	Assesses enthusiasm for upcoming pleasurable events or planned activities; the anticipation of pleasure; eagerness (interest) for doing things.
**Openness to new experience**	Assesses the openness to learning about or trying new things.
**Concern about day-to-day issues**	Assesses the concern about day-to-day issues.

### Field Testing of Interview Questions

Field testing of interview questions in prHD (UHDRS Diagnostic Confidence Level < 4) and early HD (within 5 years from onset of clinical motor signs) was conducted within the PREDICT-HD program and at independently contracted sites. All data collection sites had IRB/EC approval and all participants provided informed consent. Prior to conducting the clinical interview, all raters were trained (via webinar or in person) to ensure that all trainees had an adequate conceptual understanding for administering and scoring each of the items. A minimum sample size of 100 was targeted.

### Data Analysis 

The distribution of the composite score for each individual item was compiled, and summary statistics  associated with each item score were computed. Distributions of item scores for prHD and HD subgroups were statistically compared using the non-parametric Mann-Whitney U test.  

Non-parametric item response analyses were performed to determine the relationship between scores on the individual interview questions and total score. Item Response Theory has been demonstrated to be useful in evaluating the performance of individual items (symptoms) on rating scales, by assessing the relationship between a score assigned to an item and the overall severity of the disease[6] [7]. In order to apply IRT to early scale development work we utilize the non-parametric TESTGRAF software and IRT models[6] to generate Option Characteristic Curves (OCCs) that display the probability of a particular option score (i.e., a score of 0, 1, 2, 3, 4) on each interview question as a function of overall level of severity. In the present analyses, total item score was used as a measure of severity. To illustrate this, Figure 2 depicts a hypothetically ‘‘ideal’’ item from an item response perspective, which is characterized by a clear identification of the range of severity scores over which an option is most likely to be endorsed, rapid changes in the curves that correspond to changes in severity, and an orderly relationship between the weight assigned to the option and the region of severity over which an item is likely to be endorsed. As such, OCCs provide a graphical representation of how informative a particular item (or symptom) is as a measure of illness. Frequency distribution of option scoring within each interview question were also generated.

**Figure:**
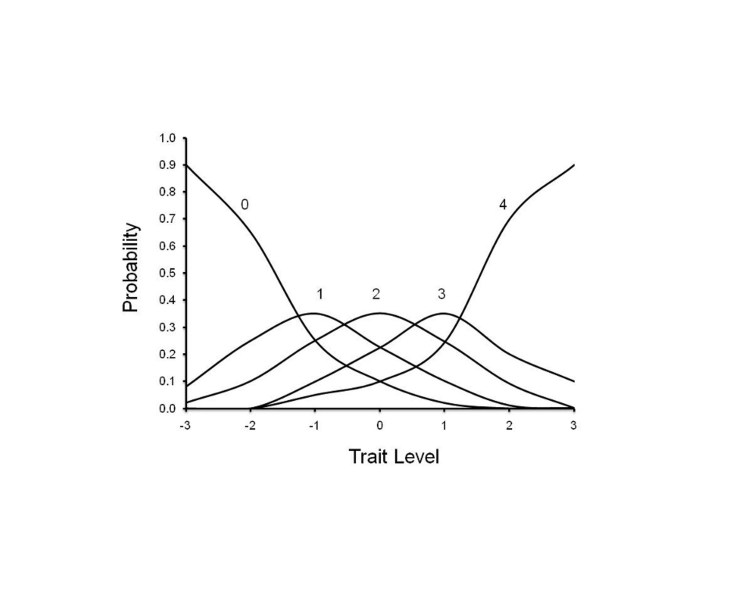
**Figure 2**. OCC for a hypothetically ‘‘ideal’’ item. The OCC shows the probability (y-axis) of endorsing a particular option for the item at different levels of the trait (x-axis)

Interview questions which were found to produce scoring across ranges of overall severity were putatively selected for further testing. Total scores for prHD and HD subjects were computed and compared statistically using the Mann-Whitney U test. The measure of internal consistency was estimated using Cronbach's alpha, and the corrected item-total correlation (between each individual item score and the total of the other selected items) was computed. Correlations between the total score and scores of individual questions not selected for further testing in the were also computed.

## Results

A total of 202 CRFs were completed (Depression and Anxiety, N=136; Apathy, N=181, Depression, Anxiety, and Apathy N=115). The participant demographic characteristics are shown in Table 2.

**Table 2.**

Demographic Characteristics 

**Table:** 

** **	** All Subjects **	** prHD**	**HD**
**Sample size**	N=202	N=115 (57%)	N=87 (33%)
**Male gender**	N=95 (47%)	N=46 (40%)	N=49 (56%)
**Age**	46.8 (18-78)	43.8 (18-78)	50.8 (23-73)

A follow-up meeting (via webinar) with the working groups was held to review data and make recommendations in moving forward, including item deletion and modification/refinement. The FDA PRO Guidance was used to guide the decision making process.[4] 

OCCs and scoring frequency distributions were generated for each of the interview questions. Of the 18 tested,  8 interview questions were found to produce scoring and discrimination across ranges of overall severity:

### ✓  Sadness

### ✓  Brightening of mood

### ✓  Drive-Initiation of routine tasks

### ✓  Drive-completion of tasks

### ✓  Lack of energy (Figure 3, as example)

### ✓  Tiredness/fatigue

### ✓  Tension

### ✓  Confidence

**

The internal consistency of these eight items was high, as were the corrected item-total correlations (Table 3, shaded rows). Cronbach's alpha was 0.89 with respect to the entire study population, 0.89 with respect to the prHD subgroup and 0.88 with respect to the HD subgroup. Among these eight items, all corrected item-total correlations were 0.52 or higher with respect to the HD subgroup, and all corrected item-total correlations (with the exception of that for the Tension item) were 0.66 or higher with respect to the prHD subgroup. 

**Table 3.** Correlations between interview Question Scores

**Table:** 

** Item **	**Item-total correlation (all subjects)**	** Item-total correlation (prHD subjects)**	**Item-total correlation (HD subjects)**
**Sadness**	0.63	0.66	0.58
**Brightening of mood**	0.68	0.68	0.71
**Drive-initiation routine **	0.71	0.68	0.77
**Drive-initiation enjoyable**	0.66	0.69	0.58
**Drive-completion of tasks**	0.68	0.73	0.60
**Lack of energy**	0.74	0.80	0.62
**Tiredness/fatigue**	0.70	0.72	0.67
**Blunting of affect**	0.44	0.54	0.13
**Ability to relax**	0.57	0.60	0.53
**Tension**	0.44	0.40	0.52
**Excessive Worry**	0.51	0.44	0.66
**Worry about HD**	0.44	0.40	0.52
**Confidence**	0.71	0.70	0.73
**Rumination**	0.59	0.63	0.54
**Self-care**	0.52	0.59	0.40
**Enthusiasm**	0.64	0.74	0.44
**Openness to new experience**	0.47	0.50	0.47
**Concern about day-to-day issues**	0.44	0.48	0.45

It was agreed that these 8 interview questions would be modified accordingly for testing in subsequent iterations; examination of the OCCs provided data on which decisions could be made as to where modifications should be made to improve item performance, including changes in wording and scoring options. For example, Figure 3 shows the OCCs for the "Lack of Energy" question. The options with the highest probably of being scored for symptom intensity increased from "0" to "1" (Mild: Some lack of energy, some things are more of an effort; some sluggishness) to "2" (Moderate: Experience of lethargy, slowness, heaviness; some reserve energy can be called upon; lots of things seem to require more of an effort). With respect to the frequency of symptoms, occasional, much of the time and almost all of the time were endorsed with increasing frequency of symptoms. Of note was the orderly progression of option scoring for the composite score, suggesting that overall severity of this symptom is best represented when both intensity and frequency are incorporated into a single option. Also of note was that the distribution of options scoring was similar in prHD and HD participants (see Figure 3, frequency distributions). The mean total composite score with respect to the above eight items was 6.43 in prHD subjects and 6.14 in HD subjects; the difference in mean scores was not statistically significant (*p* = 0.83, Mann-Whitney U test). No significant differences were noted in scoring between prHD and HD for any of the individual items, either on the basis of intensity of symptoms, frequency of symptoms or composite score. The above results suggest that these behavioral symptoms do not worsen in early HD or track with the development of early motor manifestations.[8]

**Figure:**
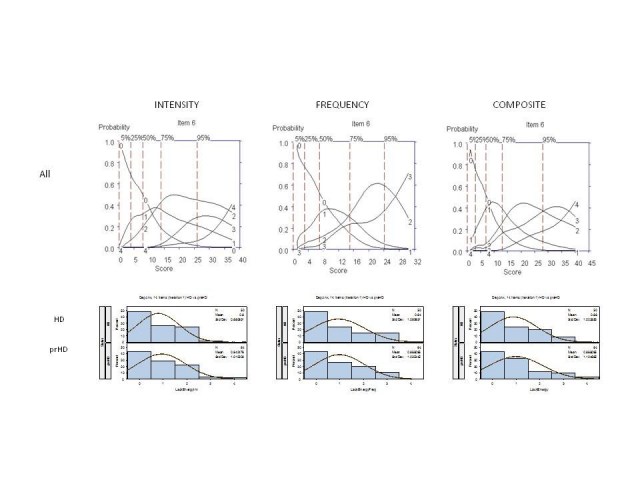
**Figure 3.** OCCs (All participants) and Frequency distribution (HD and prHD) for interview question assessing "Lack of Energy." Data are presented separately for symptom intensity (left row), frequency (middle row) and composite (right row) scores.

The remaining 10 questions had low frequency of response and poor discriminative properties, thus limiting the usefulness of these interview questions for assessment in prHD and early HD (Figure 4, as example): 

### ✗  Drive-Initiation of enjoyable tasks

### ✗  Blunted affect

### ✗  Ability to relax

### ✗  Excessive worry

### ✗  Worry about HD

### ✗  Rumination

### ✗  Self-care

### ✗ Enthusiasm

### ✗  Openness to new experience 

### ✗  Concern about day-to-day issues

There were no statistically significant differences in either the severity or frequency of symptoms between prHD and HD subjects for any of these 10 items (Figure 4, as an example). The mean composite score for each of these items was lower than the 8 questions selected for further testing, with the exception of the “Worry about HD.” For this question, an option intensity score of "1" (Mild: Worries about HD, but can be re-assured) was endorsed with the highest frequency as compared with other options, and likely reflects normal concerns associated with having HD. Indeed, when asked about "Excessive Worry" not associated with HD, scoring was low with the majority of participants endorsing option "0." Furthermore, the correlation between "Worry about HD" composite score and the total composite score from the 8 highly-endorsed interview questions was low (Table 3), and Cronbach’s alpha would not have increased upon addition of this item to the well-endorsed interview questions.

**Figure:**
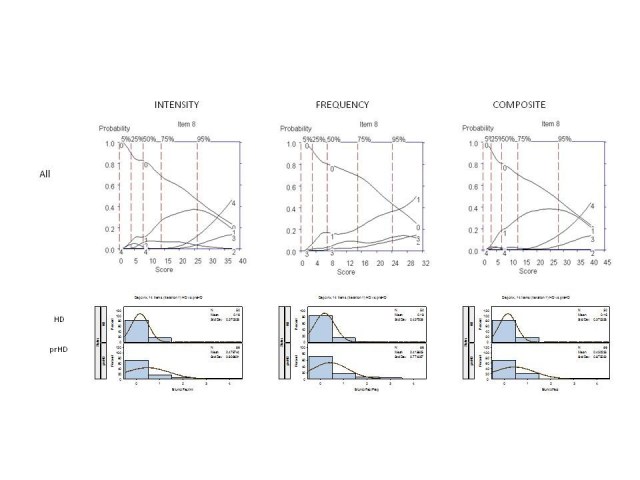
**Figure 4.** OCCs (All participants) and frequency distribution (HD and prHD) of a representative rarely endorsed interview question (Blunted Affect). Data are presented separately for symptom intensity (left row), frequency (middle row) and composite (right row) scores.

It was agreed that that these 10 interview questions should be removed from subsequent iterations on the basis of Relevance (Reported as not relevant by a large segment of the population of interest) and Response Range (A high percentage of patients respond at the floor) as outlined in Table 1 of the FDA PRO Guidance.[4]

## Discussion

FuRST-pHD has used an inclusive, iterative process to generate interview questions to assess symptoms in prodromal and early HD gene expansion carriers. While testing is still ongoing, it is clear that many CAG expanded individuals exhibit a range of behavioral changes prior to clinical diagnosis, but some symptoms are likely to be better candidates for inclusion in a final instrument than others. We report here the development and beta testing of first iteration interview questions designed to assess depression, anxiety, and apathy. Eight interview questions have been selected for further testing, have been modified accordingly by the working groups and are currently undergoing a second iteration of field testing. The results of the second iteration will be reported once completed. 

## Acknowledgments 

CHDI Foundation, Inc. – a not-for-profit research organisation whose mission is to rapidly and collaboratively discover and develop therapies that slow the progression of Huntington’s disease – initiated and sponsored the development of the FuRST-pHD. We thank Jamie Levey for her help coordinating the European focus groups, LaVonne Goodman for her help coordinating the USA focus groups, and Stacie Vik and Barbara McQuaid for administrative assistance.

## Funding Information

FuRST-pHD is funded by CHDI. PREDICT-HD is supported by the National Institutes for Health, National Institute of Neurological Disorders and Stroke (NS40068) and CHDI Foundation, Inc

## Competing Interests

The authors have declared that no competing interests exist.

## FuRST-pHD Core Team

K Anderson, B Borowsky, K Evans, J Giuliano, M. Guttman. A Ho, JS Paulsen, T Sills, A Vaccarino, D van Kammen 

## Depression and Anxiety Working Group

FuRST-pHD Core Team, D Craufurd. K Duff, M Groves, J Mundt, E van Duijin 

## Apathy Working Group

FuRST-pHD Core Team, AC Bachoud-Levi, D Craufurd. M Groves, KF Pedersen

## Statistics

S Gilbert-Evans, P Kupchak, T Sills, A Vaccarino

## Contributing Field Testing Sites

PREDICT-HD: University of Iowa, Iowa City, Iowa, USA (Leigh J. Beglinger, PhD, Tom Wassink, MD, Stephen Cross, BA, Nicholas Doucette, BA, Mycah Kimble, BA, Patricia Ryan, MSW, MA, Stacie M. Vik, BA); University of British Columbia, Vancouver, British Columbia, Canada (Lynn Raymond, MD, PhD, Rachelle Dar Santos, BSc and Joji Decolongon, MSC); Johns Hopkins University, Baltimore, Maryland, USA (Adam Rosenblatt, MD, Christopher A. Ross, MD, PhD, and Claire Welsh); Cambridge Centre for Brain Repair, Cambridge, UK (Roger A. Barker, BA, MBBS, MRCP, Sarah Mason, BSC, Rachel Swain, Anna Goodman, PhD, and Anna DiPietro); Westmead Hospital, Sydney, Australia (Elizabeth McCusker, MD, Jane Griffith, RN, David Gunn); Indiana University School of Medicine, Indianapolis, IN (Kimberly Quaid, PhD, Melissa Wesson, MS); Center for Movement Disorders, Markham, Ontario, Canada (Mark Guttman, MD, Irita Karmalkar, BA, Alanna Sheinberg, BA, and Adam Singer, BA); University of California, Los Angeles Medical Center, Los Angeles, California, USA (Susan Perlman, MD and Arik Johnson, PsyD); University of California San Francisco, California, USA (Michael D. Geschwind, MD, PhD and Jon Gooblar, BA); University of Rochester, Rochester, New York, USA (Amy Chesire, LCSW-R, MSG, Frederick Marshall, MD); Neurosciences Unit, Graylands, Selby-Lemnos & Special Care Health Services, Perth, Australia (Peter Panegyres, MB, BS, PhD, Carmela Connor, BP, MP, DP, Elizabeth Vuletich, BSC, and Steve Andrew); Washington University, St. Louis, Missouri, USA (Joel Perlmutter, MD and Stacey Barton, MSW, LCSW); Columbia University Medical Center, New York, New York, USA (Pietro Mazzoni, MD, PhD and Paula Wasserman, MA); Colorado Neurological Institute, Englewood, Colorado, USA (Diane Erickson, RN and Rajeev Kumar, MD); University of California Davis, Sacramento, California, USA (Vicki Wheelock, MD, Terry Tempkin, RNC, MSN, Nicole Mans, BA, MS); Baylor College of Medicine, Houston, Texas, USA (Joseph Jankovic, MD, Christine Hunter, RN, CCRC, and William Ondo, MD); Cleveland Clinic Foundation, Cleveland, Ohio, USA (Anwar Ahmed, PhD, Christine Reece, BS, Alexandra Bea, BA, Alex Bura, BA and Emily Newman, BA); University of Alberta, Edmonton, Canada (Wayne Martin, MD and Pamela King, RN); Clinical Genetics Centre, Aberdeen, Scotland, UK (Zosia Miedzybrodzka, MD and Daniella Rae); OCBN Contracted: Birmingham and Solihull Mental Health, Birmingham, UK (Hugh Rickards, MD, Jenny Crooks, BA, Jan Wright, BA); Center for Movement Disorders, Markham, Ontario, Canada (Mark Guttman, MD, Irita Karmalkar, BA, and Alanna Sheinberg, BA, and Adam Singer, BA); University of Melbourne, AU (David Ames, MD, Edmond Chiu, MD, Phyllis Chua, MD, Olga Yastrubetskaya, PhD, Joy Preston, Anita Goh, D.Psych, and Angela Komiti, BS, MA); University of Iowa, Iowa City, IA, USA (Leigh J. Beglinger, PhD, Tom Wassink, MD, Stephen Cross, BA, Nicholas Doucette, BA, Mycah Kimble, BA, Patricia Ryan, MSW, MA, Stacie M. Vik, BA); Huntington Disease Drug Works, Seattle, WA, USA (LaVonne Goodman, MD); North York General Hospital, Toronto. Ontario, Canada (Clare Gibbons, MS, Jeanne Kennedy, BScNEd, RN, and Wendy Meschino, MD).

## Focus Groups

EHDN Language Area Coordinators Portugal (J Ferreira, T Mestre), Spain (A Martínez Descals), France (A Durr, C Jauffret), The Netherlands (R Bos, R Roos, M-N Witjes-Ané), UK (R Fullam, O Handley, J Naji); HD Drug Works, Seattle, USA (L Goodman).

## Corresponding Author

Anthony L Vaccarino, avaccarino@ocbn.ca
